# Impact of housing environment and management on pre-/post-weaning piglet productivity

**DOI:** 10.1093/jas/skac142

**Published:** 2022-06-16

**Authors:** Brett C Ramirez, Morgan D Hayes, Isabella C F S Condotta, Suzanne M Leonard

**Affiliations:** Department of Agricultural and Biosystems Engineering, Iowa State University, Ames, IA 50011, USA; Department of Biosystems and Agricultural Engineering, University of Kentucky, Lexington, KY 40546, USA; Department of Animal Sciences, University of Illinois Urbana-Champaign, Urbana, IL 61801, USA; Department of Animal Science, North Carolina State University, Raleigh, NC 27695, USA

**Keywords:** farrowing, nursery, physiology, swine, temperature, ventilation

## Abstract

The complex environment surrounding young pigs reared in intensive housing systems directly influences their productivity and livelihood. Much of the seminal literature utilized housing and husbandry practices that have since drastically evolved through advances in genetic potential, nutrition, health, and technology. This review focuses on the environmental interaction and responses of pigs during the first 8 wk of life, separated into pre-weaning (creep areas) and post-weaning (nursery or wean-finish) phases. Further, a perspective on instrumentation and precision technologies for animal-based (physiological and behavioral) and environmental measures documents current approaches and future possibilities. A warm microclimate for piglets during the early days of life, especially the first 12 h, is critical. While caretaker interventions can mitigate the extent of hypothermia, low birth weight remains a dominant risk factor for mortality. Post-weaning, the thermoregulation capabilities have improved, but subsequent transportation, nutritional, and social stressors enhance the requisite need for a warm, low draft environment with the proper flooring. A better understanding of the individual environmental factors that affect young pigs as well as the creation of comprehensive environment indices or improved, non-contact sensing technology is needed to better evaluate and manage piglet environments. Such enhanced understanding and evaluation of pig–environment interaction could lead to innovative environmental control and husbandry interventions to foster healthy and productive pigs.

## Teaser

This comprehensive review of past and recent literature discusses the complexity and impact of the thermal environment and associated husbandry practices on the success of young pigs. Emphasis is placed on synthesizing factors associated with an optimum environment during key stages life (creep area and nursery) and during/after stressful events (i.e., farrowing, weaning, transport, and placement).

## Introduction

The environment experienced by young pigs in modern, intensive housing systems is a complex, interconnected nexus of conditions that shape the pig’s future growth, development, welfare, and health. An indoor environment can be comprised of thermal, air quality, illuminance, noise, enrichment, housing, and social components that constantly surround the pig throughout its development. For young pigs, the optimum environment cannot be prescribed by a standard set of environmental conditions (e.g., temperature, gas concentrations, lumens, decibel levels, etc.); however, it is better defined by the combination of conditions relevant to a particular housing and husbandry style that best compliments feeding, nutrition, water, genetic potential, health status, etc. Both environment and husbandry change drastically from farrowing through the first 8 wk and this transition is coupled with the stress of birth, weaning, transportation, and placement; therefore, to ensure maximum survivability, vigor, and performance are to be achieved, the diverse and complex nature of the environment surrounding young pigs requires a comprehensive evaluation and understanding.

Specific environmental conditions are often difficult to directly associate with poor performance, increased mortality, or an increased risk for health challenges, with thermal stress being an exception. However, it is often noted that the environment can exacerbate deficiencies in nutrition, low bodyweight, feed intake, mobility, etc. Seminal works, Mount and Ingram (1965), Holmes and Mount (1967), and Mount (1968), demonstrate the importance of a carefully tailored thermal environment for young pigs, which has since set the precent for many modern housing materials and management. More recently, reviews by Villanueva-García et al. (2021), Gebhardt et al. (2020), and Tucker et al. (2021) summarize the numerous factors that are associated with young pig viability, with environmental management being a key contributor and integral aspect. However, there is limited comprehensive information regarding the environment and associated husbandry practices used to enhance young pig viability.

This review will predominately discuss the thermal environment aspects (e.g., temperature, relative humidity, airspeed, etc.) and environmental management (e.g., housing, supplemental heat, etc.) of young pigs (within the first 8 weeks of life) in modern, intensive production systems, as they relate to growth, health, and mortality. Our objective is to summarize the current state of knowledge regarding environmental factors contributing to the success of young pigs, focusing on pre-weaning (creep area) and post-weaning (nursery and finishing) housing, as well as sensing technology to improve the understanding and management of the young pig–environment interaction.

## Basic Principles of the Thermal Environment

**Figure 1.**
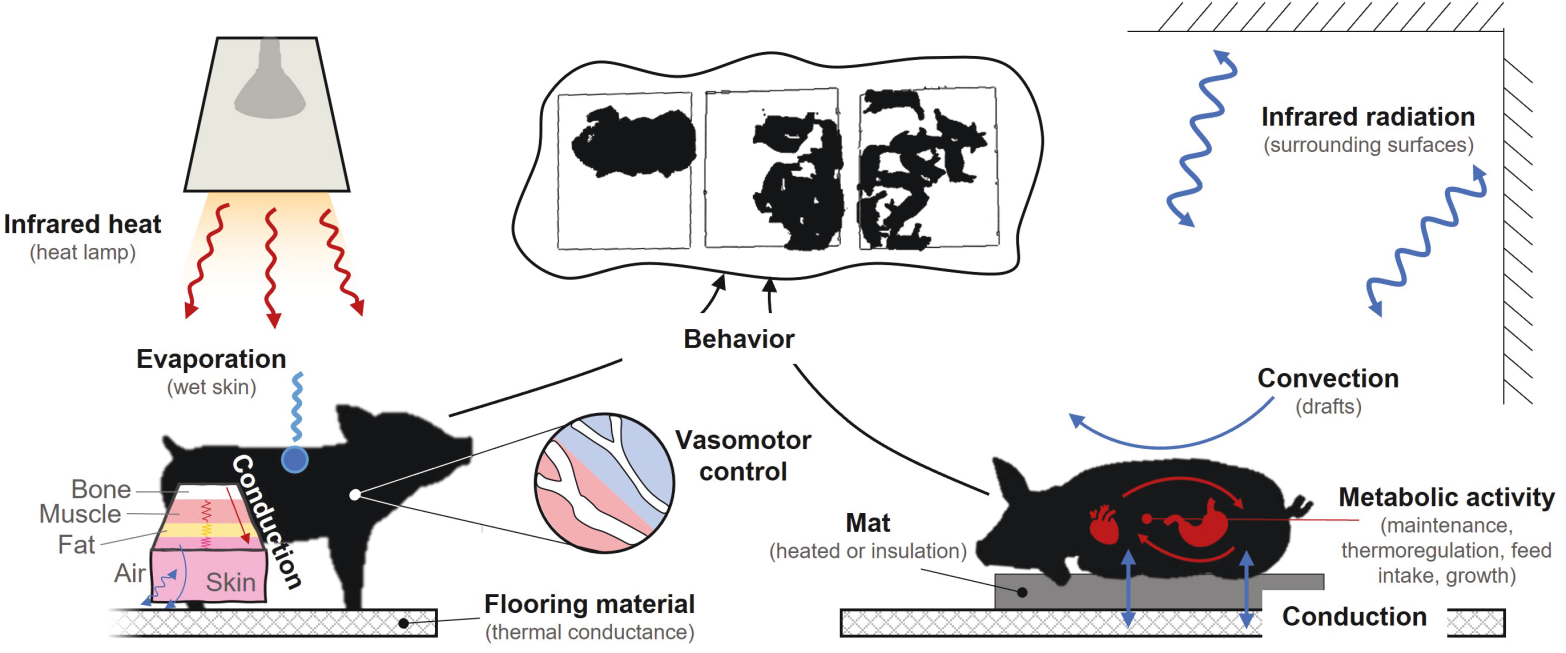
Illustration of the various heat exchange mechanisms and thermoregulation capabilities of young pigs in different environments.

The thermoregulation and thermal exchange of the young pig to its environment is multifaceted, as illustrated in Figure 1, during pre-weaning (i.e., creep areas) as well as post-weaning in either nursey or wean-finish phases. There are numerous animal and environmental characteristics that influence the rate of energy (heat) transfer to and from a pig (DeShazer et al., 2009). Hence, control of heat exchange to avoid thermal stress, which decreases energy available for growth and vigor, is paramount for productivity and livelihood (DeShazer and Yen, 2009). Pigs are homeothermic animals that use physiological and behavioral controls to maintain a near-constant body temperature (Mount, 1973). The thermal energy balance of the pig is maintained such that energy input through metabolic activity equals heat loss to the environment. Heat loss is by sensible modes of convection, conduction, and radiation, and by latent modes of water evaporation through respiratory exchange and minimally, the skin (unless wet).

### Thermal exchange and responses

In response to a changing environmental temperature, the pig initially adjusts its surface temperature through physiological processes (e.g., vasodilatation or vasoconstriction) and by adjusting its exposed skin surface area through behavioral adjustments (e.g., recumbent vs. sternum resting posture; Hillman, 2009). These responses maintain a relatively constant sensible heat loss. In warming environmental conditions, the temperature gradient between the pig’s surface and environment decreases, thereby reducing sensible modes of heat loss. In response, the pig will increase latent heat loss (via evaporation) to balance for the reduced sensible heat loss.

Young pigs are unique compared with heavier, more mature animals in such that they lack vasomotor control to regulate conductance of heat from their core body to surface, are hairless and lack subcutaneous muscle and fat, as well as have a high surface area to volume ratio resulting in high heat loss and increased susceptibility to chilling (Herpin et al., 2002). Heath (1983) showed that weaned pigs reared at 35 °C had more subcutaneous fat while those reared at 10 °C had more fat in their abdominal tissues and muscles. Weaver and Ingram (1969) also found that weaned pigs reared in a cold environment (5 to 10 °C) had more hair and were shorter and stockier, with a reduced surface area to body weight ratio, compared with those reared at warmer temperatures (35 °C).

### Driving forces for heat exchange

A temperature (i.e., air, contact, and radiant) and/or water vapor pressure gradient must exist to drive heat exchange between the pig and its surrounding environment. A positive gradient results in a heat gain while a negative gradient results in a heat loss. The rate of heat loss can also be modified by thermal conductance (e.g., vasomotor control or flooring material) and air velocity (e.g., heat loss increases with increasing air velocity). These gradients and modifiers can be beneficial or detrimental depending on the size, housing, health, etc., of the pig (DeShazer et al., 2009). An understanding of how these gradients and modifiers influence the rate of heat loss and how to assess them is imperative for improving the productivity and livelihood of young pigs.

The main descriptor of the environment is air temperature; albeit it only directly affects the convective heat loss of the pig. Prudence is needed when using exclusively air temperature to describe and assess the thermal environment. An effective representation of the thermal environment can be achieved by combining air temperature with measurements representing the other driving forces and modifiers. Contact temperature of flooring influences conductive heat loss and is important as pigs spend considerable time lying on the floor; however, analysis of the heat loss from the pig to the floor is complex as the contact temperature varies with internal thermal conductance via vasomotor control of the pig and material properties of flooring. Surface temperatures of a pig’s surroundings can vary considerably and the presence of a skin to surface temperature gradient drives thermal radiation heat exchange. For example, floors, walls, ceiling, and supplemental heat sources (e.g., heat lamp) can all at be different temperatures. Finally, a vapor pressure gradient, that is, the difference in vapor pressure between the skin or respiratory tract and the ambient air, influences the rate of cutaneous or respiratory evaporation. As moisture evaporates, heat must be removed from the source (either skin or respiratory tract) resulting in heat loss. Wet skin (e.g., from birth fluids, wallowing, sprinklers, etc.) increases this gradient; therefore, greater cutaneous evaporation and heat loss.

Air velocity is a modifier to both temperature and vapor pressure gradients as it effects convective heat loss as well as evaporation rate from wet skin. As air velocity increases, the rate of convective and evaporative heat loss increases at diminishing rates. An additional modifier, thermal conductance, influences the rate of heat loss by conduction and can modify internal heat exchange (i.e., core to skin via vasomotor control and body composition) and to the environment (i.e., skin to contact material via thermal properties of the material or flooring).

## Pre-weaning Piglets

The pre-weaning phase inherently involves numerous stressors including, but not limited to birth, thermal stress shortly after farrowing, competition for colostrum, social stress of establishing litter hierarchy, and the environmental stressors of the creep area. This section will focus on the environmental stressors associated with farrowing and housing of pre-weaned pigs while recognizing that health and social stressors interact strongly with the environmental stressors.

### Climate physiology and energetics

Newborn piglets are highly susceptible to chilling and hypothermia. Piglets experience a significant reduction in environmental temperature when they are expelled from the 38.8°C sow into a farrowing room with air temperature typically around 22.5°C. Piglets are born wet with an average of 28.8 g of birth fluid moisture on their surface (Christison et al., 1997), which quickly evaporates, thereby cooling the piglet and further challenging the piglet’s ability to maintain its body temperature. Low piglet birth weights exacerbate thermoregulatory challenges and subsequently increase pre-wean mortality rates (Zotti et al., 2017; Feldpausch et al., 2019).

To compensate for their poor thermoregulation abilities, piglets use behaviors such as shivering, seeking heat sources, and huddling with other litter mates and the sow (Villanueva-García et al., 2021). Young piglets, especially in the first week of life, use posture changes more than the degree of huddling with littermates to thermoregulate (Vasdal et al., 2009). Piglets prefer to spend the greatest proportion of time near the sow in the first few days of life regardless of type and location of microclimate areas or room temperature, so it is likely that there are multiple biological factors involved, not solely heat seeking behavior (Hrupka et al., 1998; Vasdal et al., 2010). The risk of hypothermia for piglets decreases throughout lactation as they develop body fat and thermoregulation abilities.

### Environmental management: microclimates

The primary way to manage the environment for pre-wean piglets is to provide a small area of the farrowing stall that better meets the thermal needs of the piglets during lactation, called a microclimate. Microclimates reduce utility cost and sow heat stress compared with warming the entire farrowing room to meet piglet needs. The key elements of creating a piglet microclimate are supplemental heat source type and adequate space allocation.

Commercially available microclimate heat sources are generally of 2 types: conductive (mats or in-floor heat) or radiative (heat lamps or plates). Radiative heat sources, especially heat lamps, are typically suspended over a rubber or insulated mat on the floor of the creep area to provide a more comfortable and uniformly heated area for the piglets. Multiple authors report no difference in piglet weight gain or pre-wean mortality when comparing farrowing stalls with heat lamps or heat mats, although heat mats are 32% to 73% more energy efficient than lamps in field-scale comparison studies (Besheda et al., 2014; Stinn and Xin, 2014; Lane et al., 2020). Piglets have been observed to show preferences in heat source type. In the critical first days after birth, piglets prefer radiative light bulbs over incandescent light bulbs (Larsen et al., 2017) and heat mats (Zhang and Xin, 2001). Larsen and Pedersen (2015) reported that piglets prefer to sleep in dark areas of the farrowing stall at night beginning at 3 d of age, so using mats or non-illuminating heat lamps could prove advantageous in mid and late lactation.

Partially or fully enclosed microclimates are increasing in diverse designs and features. Partitions on the top and sides of the heating element can provide more uniform conditions inside the microclimate, decrease wasted heat to the room environment, and reduce potential drafts on the piglets. Smith et al. (2019) reported a 50% reduction in microclimate heating element electricity usage and a 2% reduction in over-lay mortality when a semi-enclosed radiatively heated microclimate was used compared to a traditional heat lamp; however, no significant differences were found in overall pre-wean mortality or average daily weight gain. In an open pen farrowing system, Vasdal et al. (2010) reported that piglets spent equal amounts of time in open and enclosed creep areas with sawdust bedding, suggesting that piglets do not exhibit a preference between open and partially enclosed microclimate areas.

With industry trends of increasing number of piglets born live (PigChamp, 2021), providing adequate microclimate floor area is critical to accommodate all piglets in the litter. Based on a wean weight of 8.6 kg, Wheeler et al. (2007) recommended 0.091 m^2^ of thermoneutral floor space per piglet. A more recent study by Smith and Ramirez (2021) suggested slightly less space is required, at 0.080 m^2^ per 8.6 kg piglet based on a cylindrical approximation. When provided two or one heat lamp, piglets spent on average 4 h more per day in the heated creep area of two heat lamps, though there was no difference in piglet survivability or weight gain (Leonard et al., 2021). This suggests that there is no productivity advantage to an oversupply of heated creep area. Piglets require mat temperatures between 30 and 44.5 °C to meet their thermoneutral needs and due to the shape and configuration of the heating element, not all of the heated microclimate area may be within the desired temperature range (Davis et al., 2008). Thermal imaging can determine the quantity of heated area that is within the useful temperature range for the piglets.

### General environmental management

Piglets are most susceptible to hypothermia in the first 2 h after birth, and much research has targeted intervention options to limit the magnitude and duration of piglet body temperature drop during this period. Flooring type influences piglet temperatures, with metal flooring reducing piglet rectal temperature drop compared to solid concrete flooring (Pedersen et al., 2016) and plastic-coated expanded metal flooring as a better alternative to concrete slats for improved piglet performance (Stansbury et al., 1987). In an experimental setting, Pedersen et al. (2016) determined that straw bedding was more effective than suspended or on-floor radiant heating plates at limiting rectal temperature decline after birth for undried piglets; however, regional differences in production may render straw bedding as unfeasible. Regardless of flooring type, providing a comfortable insulated surface in the piglet resting area will reduce conductive heat loss of the piglets.

Microclimates are commonly placed beside the sow to accommodate suckling piglets. However, placing microclimates near the rear of the stall can improve piglet usage in the most critical first 2 d of life (Zhang and Xin, 2001). Andersen and Pedersen (2015) found that radiative heating plates mounted above the rear of the farrowing stall increased the time newborn piglets spent in the rear zone of the stall by an average of 4 min and reduced piglet rectal temp drop in the first 4 h after birth without impacting time to first udder contact or time to first suckle, providing further evidence that heat sources at the rear of the stall during farrowing are advantageous. Hrupka et al. (1998) found no differences in piglet survival when heat lamps were placed beside or in front of the sow stall.

Another method to reduce the likelihood of hypothermia for piglets is to raise room setpoint temperature to 25 °C or greater during farrowing (Pedersen et al., 2013; Vande Pol et al., 2021). However, elevated room temperatures during farrowing can lead to poor sow performance or mortality (Stansbury et al., 1987). Beginning on the day after farrowing, room setpoints should be gradually reduced to 18.8 °C by 7 to 10 d (PIC, 2017) to reduce utility usage and heat stress on the sow. Further, Stansbury et al. (1987) found that maintaining room temperatures of 30 °C throughout lactation increased preweaning mortality and reduced piglet weight gain, highlighting the close relationship between sow and piglet performance.

### At-birth intervention strategies

In addition to passive environmental conditions to improve newborn piglet outcomes, at-birth caretaker interventions are used to reduce the heat loss and subsequent piglet rectal temperature drop immediately after birth due to drying of amniotic fluid. Vande Pol et al. (2020a) reported that drying piglets with a cellulose-based desiccant was more effective than drying with paper towels to limit rectal temperature drop, with either drying method being better than no intervention. Placing piglets in a warming box under the heat lamp for 30 min after birth is better than no intervention but not as effective as actively drying the piglets, with best results produced from both drying and warming piglets (Vande Pol et al., 2020b). While drying reduces rectal temperature drop, Christison et al. (1997) reported that the vigorous stimulation piglets receive when being rubbed while drying does not provide an advantage in getting piglets to nurse sooner compared to gently placing the piglets under heat lamps.

Despite minimizing the rectal temperature drop of neonatal piglets, drying and/or warming piglets had no effect on piglet wean weight or pre-wean mortality over the entire lactation period (Vande Pol et al., 2021), highlighting that pre-wean mortality is a complex, multi-faceted problem and not solely a product of hypothermia at birth. Vande Pol et al. (2021) reported 74.3% of variation in pre-wean mortality was explained by piglet birthweight, with piglet rectal temperature 30 min after birth not being of practical importance. These collective works report that drying and warming boxes are most effective for light piglets (birth weight less than 1.5 kg) and cooler farrowing room temperatures (less than 25 °C). The majority of pre-wean mortality can be attributed to a combination of chilling, malnutrition, and crushing, so preventing hypothermia at birth may be one of many interventions needed to improve piglet survivability.

### Practical considerations

Provision of a comfortable microclimate is proven effective in reducing pre-wean mortality. Regardless of supplemental heat source choice for the piglet microclimate, ensuring correct operation is critical. Visible light spectrum heat lamps provide obvious visual cues when operational, but it can be more challenging for caretakers to assess if heated mats are functioning properly. Visual inspection of piglet posture and huddling patterns or a mat “touch test” can be subjective, so using a handheld infrared thermometer to confirm correct operation is recommended.

Programmable thermostats for mats and enclosed microclimates, or adjustable heights for lamps, are helpful for accommodating the changing thermal needs of growing piglets. Additionally, adjustable thermostats can reduce utility usage and prevent unnecessary overheating of the farrowing room if room temperature approaches microclimate set points.

Partially enclosed or flexible sided microclimate nests can reduce air drafts on piglets, but can also present additional husbandry challenges. It is difficult to visually assess all piglets when they are under a partition, even if there is a transparent cover. Some covered microclimates feature a pulley system so the caretaker can raise the cover and evaluate the piglets beneath, but this involves additional time and labor. Further, the enclosures can create obstacles for caretakers when catching piglets for husbandry practices.

Everything in the farrowing environment must be cleaned and disinfected between groups of sows. Heat lamp bulbs break easily when in contact with water and are therefore typically removed from the farrowing room prior to wash down then re-installed for the next farrowing. Mats must be inspected for damage, disinfected, and thoroughly dried between litters. Many enclosed microclimates are not designed to be removed between batches; rather, they must be cleaned and disinfected in place.

## Post-weaning Piglets

The weaning phase inherently involves numerous stressors including, but not limited to the nutritional stress of transitioning from milk to a grain-based diet, social stress of removal from the sow, transport stress involved in moving these young pigs to new facility, social stress of a new social hierarchy as mixing of litters occurs, and environmental stressors in this new facility (Hötzel et al., 2011). This section will focus on the environmental stressors associated with both the transport and housing of weaned pigs while recognizing that nutritional and social stressors interact strongly with environmental stressors.

### Climate physiology and energetics

While newborn piglets have poor thermoregulatory abilities, by the time weaning occurs the ability of the pigs to thermoregulate has improved. These young pigs have established a layer of fat to provide some insulation as well as developed an intake energy level that allows for metabolic processes and growth to occur, providing a level of heat production by the pigs that can be utilized for thermoregulation. However, the shift in diet can strongly affect the heat production by the pigs due to metabolic energy intake changes (Le Dividich and Herpin, 1994). When these pigs are weaned the energy intake through feeding is generally low for a number of days (Bruinix et al., 2002; Van der Meulen, 2010). Without this energy input, pigs are often in a negative energy balance for 4 to 6 d after placement in their new pens (Le Dividich and Herpin, 1994).

The age at weaning also affects how these young pigs can respond to these stressors in this weaning period. Early weaning, in this case defined as prior to 20 d of age, inherently means the young pigs have not established a substantial layer of fat. Without this subcutaneous fat to provide insulation, pigs are more susceptible to cold stress. For a pig weaned at 2 wk of age, the negative energy balance caused the young pig to lose 25% to 30% of its backfat in the first week following weaning (Fenton, 1985). It has also been shown that it takes 4 to 6 wk for the backfat to be recovered (Seve, 1982). Until these young pigs have positive energy balances allowing for adequate heat production, a byproduct of the metabolic processes, growth, and fat deposition to begin, it is not practical to start reducing air temperatures for these pigs.

Thermoneutral conditions for weaned pigs are fairly consistent among published recommendations with studies tending to focus on the lower critical temperature or the minimum room temperature for these young pigs as cold stress tends to be the primary concern. Studies have recommended the lower critical temperature is between 26 and 28 °C for the first week following weaning (Le Dividich and Herpin, 1994). The next 2 wk the lower critical temperature is near 24 °C and then each subsequent week, a 2 to 3 °C reduction can occur until typical finishing conditions are achieved (Le Dividich and Herpin, 1994). Commercial suggestions agree with a range of comfortable temperatures from 18 to 27 °C for nursery pigs over 14 kg and 27 to 32 °C for weaned pigs under 14 kg (NPPC, 2003).

The two other primary environmental conditions that interact with air temperature to affect the thermal environment experienced by the pigs are relative humidity and airspeed. In general, humidity is not known to strongly influence pig’s environmental responses, if the temperatures are maintained within thermoneutral conditions (Le Dividich and Herpin, 1994). However, Georgiev et al. (1977) found relative humidity levels of 50% and 90% showed significant differences in heat production of smaller 23 kg pigs at the extreme temperatures of 5 and 35 °C. Weaned pigs are fairly susceptible to elevated airspeeds. When given the ability to select temperature by turning on a heat lamp, weaned pigs show a strong correlation between air speed and air temperature preference with an air temperature preference of 17.9 °C at an airspeed of 0.08 m/s versus a temperature preference of 21.7 °C at an airspeed of 0.4 m/s (Verstegen et al., 1987).

### Impact of environment: transportation

Transport stressors include the mixing of litters, overcrowding, noise, vibrations, hot and cold temperatures, as well as temperature fluctuations. While transport is necessary to move weaned pigs from farrowing rooms to either nursery or wean-finish barns, the decisions made on how to stock the trailer, how far to transport the young pigs, and what type of trailer to use all affect how these pigs are influenced by transport stresses (Roldan-Santiago, 2013). Longer duration transports and higher temperatures have been shown to exacerbate stress and increase risks for dehydration in transport (Wamnes et al., 2008). Berry and Lewis (2001) identified a statistically significant reduction at day 3 and observed 1 wk reduction in feed intake for weaned piglets transported at 35 °C for 24 h compared with shorter transport durations of 0 or 6 h. Transport mortality rates have been correlated with warm ambient conditions and cold ambient conditions, with the greatest mortality rates associated with warm ambient conditions (>25 °C), followed by cold ambient conditions (<15 °C) during transport. The lowest transport mortality rates have been correlated with moderate ambient conditions between 15 and 25 °C (Zhao et al., 2016). In the same study, when evaluating the interaction of warm conditions (warmer than 25 °C) and transport duration, each increase in transport duration (<600 km, 600 to 900 km, 900 to 1,200 km, 1,200 to 1,500 km, and > 1,500 km) led to a significantly higher predicted mortality rate. Warm conditions and greater than 1,500 km had a predicted mortality rate of greater than 0.4% compared with the measured 0.0333% across all temperatures and transport durations (Zhao et al., 2016). Similarly, pig ear surface and rectal temperatures also reflect differing ambient transport conditions demonstrating potential shifts in body temperature. In warm conditions, ear and rectal temperatures of 36.2 and 39.2 °C have been recorded, while ear and rectal temperatures during cold conditions were 23.1 and 38.6 °C, respectively (Lewis, 2008).

In addition to both the temperature and duration of transport, stocking density in the trailer also influences mortality rates. An increased stocking density allowing only 0.05 m^2^/weaned pig compared to typical stock density which allows 0.06 to 0.07 m^2^/weaned pig resulted in an increase in lesions, stress indicators in blood samples, and more pigs piling on other pigs (Sutherland et al., 2009). However, Harmon et al. (2017) did not find a relationship between stocking density and compartment temperature, indicating the stocking density concern is not causing thermal stress but perhaps, a social stress or an inability to settle comfortably, is causing lesions or fatigue (Sutherland et al., 2009).

### Impact of environment: facility

When evaluating responses to the environment it is important to note if the studies were conducted in the first or second week following weaning. In the first week, pigs are struggling to meet nutritional requirements and recover from the negative energy balance they experienced during weaning, as opposed to later in the nursery phase after the pigs have adapted to a solid diet (Nienaber, 1985). Riskowski and Bundy (1990) found that in the 2 wk after weaning, daily gain was affected by airspeed, with greater weight gains associated with lower airspeeds and feed intake was found to increase with decreasing temperatures. Overall results of the study indicated that maintaining the lowest airspeeds possible is important to getting the best performance on these pigs initially following weaning. While the published thermoneutral conditions agree with these findings, the study indicates that a slight decrease in air temperature may be acceptable, if low airspeeds can be maintained (Riskowski and Bundy, 1990).

As feed intake is more established, pigs weaned for two or more weeks still have limited feed intake. It has been observed that as the temperature decreases below the pigs thermoneutral zone, their feed intake will increase slightly but as temperatures continue to decrease, the feed intake will plateau and even decrease slightly (Quiniou et al., 2000). This limited feed intake is extremely important to understand why cold stress is such a concern with young weaned pigs. Cold stress on older pigs can create some inefficiencies in feed conversion due to increased feed intake; conversely, in weaned pigs, it leads to morbidity as well as agonistic behaviors. Both vocalizations and infrared images of surface temperature have been tested for monitoring cold stress with limited success (Cordeiro et al., 2018; da Silva et al., 2019; da Fonseca et al., 2020). While the monitoring is not as reliable as desired, the stress responses themselves are consistently measured and indicative of performance concerns.

It is important to note that while cold stress tends to be the focus of nursery management studies, it is also possible to heat stress weaned pigs. A study by Ferrari et al. (2013) examined an upper critical temperature of 29 °C and found that these weaned pigs increased both vocalizations by 80% and respiration rate from 33 to 116 breaths per minute as temperatures went from 29 to 41 °C. Also, the primary concern with high relative humidity is related to warm temperatures where high humidity affects the physiologic responses of the pigs attempting to adapt to the warm conditions. For instance, an increase from 22 to 28 °C caused a 25% decrease in growth rate at 50% relative humidity and when relative humidity increased from 50% up to 90% at 28 °C an additional 8% decrease in growth rate was identified (Morrison, 1969).

### Other housing factors

Floor type varies substantially in the weaning phase with slatted concrete, solid concrete, woven metal, plastic coated metal floors, as well as bedded pens all included in various studies. It is evident that flooring strongly influences the pig’s interaction with their thermal environment. A bedded floor provides the most insulation and therefore allows for lower air temperatures while warmer air temperatures are needed to balance greater conductive heat loss with the floor for flooring materials with high thermal conductance (e.g., metal). Mount (1975) found a bedded solid floor created an effective temperature comparable to a 3.9 °C warmer room temperature without bedding. Slatted floors are the most common flooring seen in the swine industry due to cleanliness, reduced labor, and improved manure management, and plastic-coated metal floors have been shown to reduce foot lesions, leading to better weight gain (Lindemann et al., 1985). However, the materials used to create the slatted floor do affect the effective temperature experienced by the pigs. Morrison et al. (1987) found that compared to a bedded floor, a solid concrete floor had an effective temperature that was 2.8 °C cooler, a rubber coated metal floor was 3 °C cooler, and a solid metal floor was 5.8 °C cooler. While there is very little data on the effective temperature of concrete compared with plastic coated metal slatted floor for weaned pigs, it has been shown that sows are able to dissipate more heat on a concrete floor compared to a plastic-coated metal floor (McGlone et al., 1988), suggesting that a plastic-coated floor may provide slightly less heat loss compared to a concrete floor.

Stocking density also influences how pigs experience their thermal environment. Huddling is an effective behavior to reduce heat loss by reducing surface area in contact with air or flooring surfaces instead having that surface area in contact with other pigs. As group size increased from 1 to 4 pigs, temperature preference shifted from 30.2 to 20.2 °C (Robbins et al., 2021). However, in a similar study moving from 1 to 4 pigs per pen, 1.44 to 0.35 m^2^/pig, respectively, in the 4 wk after weaning with thermoneutral conditions, individual pigs gained 16 kg compared with the 14.5 kg gained by the group of four (Spicer and Aherne, 1987). Both weaning stress and maintaining too high of a stocking rate are also known to be associated with some aggressive behaviors. Laves et al. (2021) tested an option of adding a second level to pens to increase floor space for each pig in the weaning period. This shifted the space allowance from 0.38 m^2^/pig without the raised platform to 0.45 to 0.51 m^2^/pig with the raised platform. The increased space in combination with the ability for pigs to avoid line of sight with other pigs led to an increase in weight gain as well as reduced skin injuries and observed fighting (Laves et al., 2021). Commercial recommendations for stocking density call for at least 0.3 m^2^/pig (MWPS, 1997). There are clearly some benefits to increased spacing allowance, but there is a potential tradeoff with thermal management.

## Sensing Technology for Research and Management

The continuous monitoring of environmental conditions is critical to achieve high production efficiency, sustainable operation, and bridge the gap between targeted and actual production environment. Precision livestock farming uses technology to provide animal caretakers with tools that allow continuous real-time monitoring of the production system (Berckmans, 2017). These tools reduce human workload and empower producers to efficiently use their time and make well-informed management decisions based on actual animal and environmental status versus infrequent comparison to generic standards or tabulated values. These standards have become obsolete since they do not account for the latest advancements in animal genetics, nutrition, and management practices, which have led to substantial shifts in the environmental needs in intensive housing (Fournel et al., 2017).

**Figure 2.**
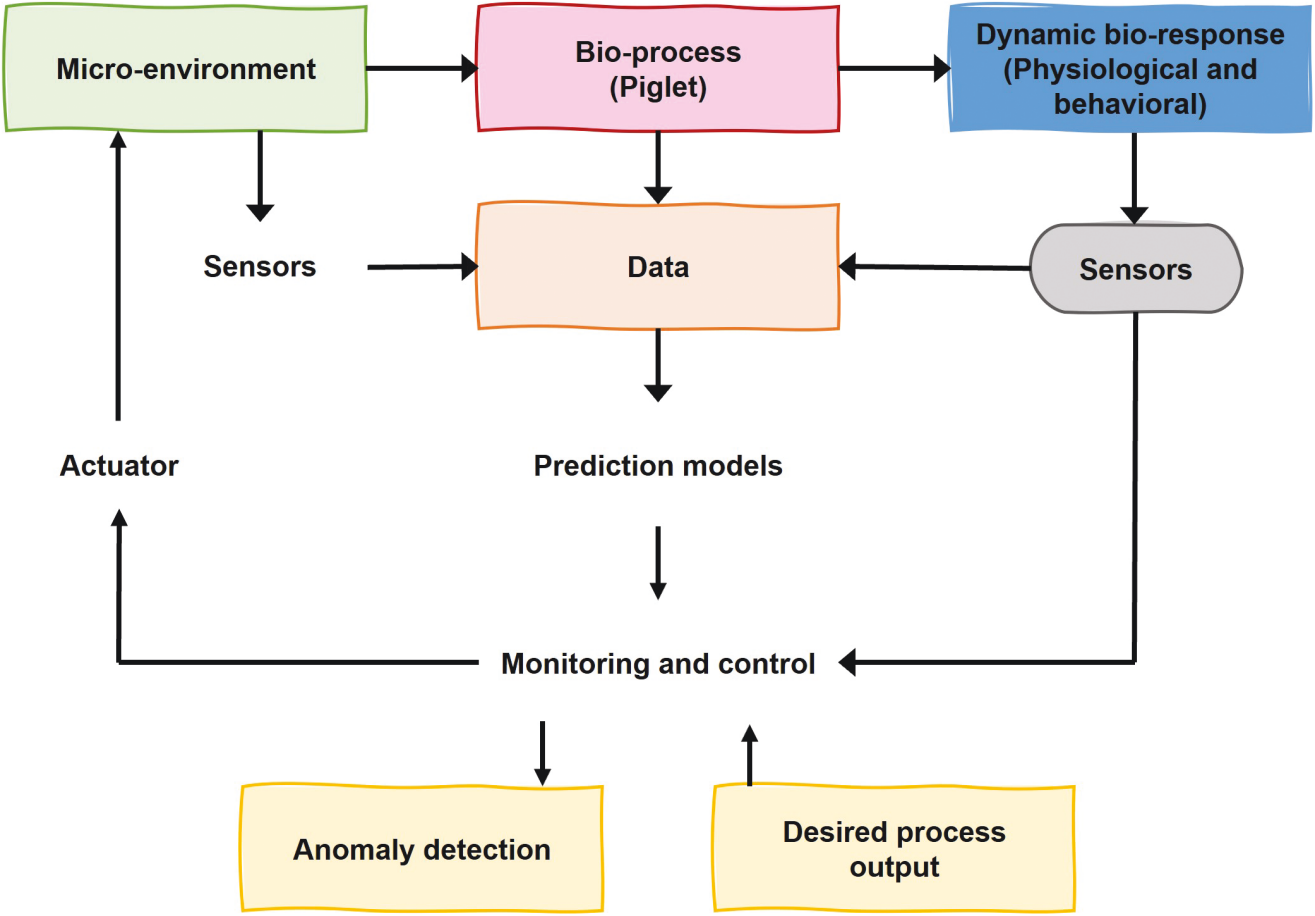
Schematic overview of a precision livestock farming system. The environment control is based on physiological and behavioral animal responses (adapted from Aerts et al., 2003 and Fournel et al., 2017).

An alternative approach to environmental management could be based on the animals’ physiological and behavioral responses to the environment for more reliable performance and, potentially, increased profitability (Black and Banhazi, 2013). For such a system to work, four conditions should be fulfilled (Berckmans, 2006; Wathes et al., 2008): (1) real-time monitoring of animal behavioral or physiological state through sensor technology, (2) a reliable, continuous prediction (expectation) of animal variables based on bioresponse and bioenergetic models, (3) a predetermined target value for the animal variables (e.g., based on animal comfort indices), and (4) data integration between predictions and real-time measurements for automatic monitoring and/or management. Figure 2 shows a schematic overview of such a system.

Environmental control of the swine facilities requires appropriate instrumentation to collect information on both the environment and the animals’ behavioral and physiological responses. Commonly monitored piglet and environmental variables, as well as a description of sensors to promote precision farming are described below. These are not exhaustive but show the possibilities of using both animal response and the environment as feedback to create a robust monitoring and control system (Figure 2).

### Physiological measurements

Piglet body temperature is an important variable as it is linked to health and thermal status. Continuous body temperature measurement in commercial settings is a challenge as currently developed methods have been developed for research purposes, such as rectal/tympanic probes and implanted sensors (Eigenberg et al., 2009). These are too invasive and complicated to apply on a commercial scale. Thermal imaging is, perhaps, the most promising sensing technology for continuous piglet skin temperature assessment in commercial farm environments.

Two approaches to determine the health status of piglets from thermal imaging have been evaluated. The first approach correlates the pig’s superficial temperature with its core body temperature through different modeling approaches (Loughmiller et al., 2001; Warriss et al., 2006; Chung et al., 2010; Mostaço et al., 2015; Xiong et al., 2018). The body locations that presented the best correlation with core body temperature are considered thermal windows, such as the ear base and eyes.

Because pigs’ superficial temperature varies with environmental temperature (Andersen et al., 2008), the second and more reliable approach to assess piglets’ health status would be to detect a sudden and remarkable change of superficial temperatures. Lu et al. (2018) demonstrated the feasibility of automatically extracting ear base temperature from thermal images, which can enable continuous body temperature assessment in commercial settings.

Respiration rate is an indicator of thermal stress as it correlates with dry-bulb temperature (Brown-Brandl et al., 1998). Because it has little lag time relative to dry-bulb temperature, it promptly reflects the animal’s thermal status (Eigenberg et al., 2009). However, continuous automatic respiration rate measurement in a commercial production system can be challenging, as the most commonly used method to acquire this variable is to count flank movement over a period of time. This method is not only laborious but can also be flawed as the human presence to observe the animals can generate changes in respiration rate. An effort to develop an automatic respiration rate sensor based on sound analysis was made by Eigenberg et al. (2002), but the need for pigs to wear a vest makes this approach unfeasible in commercial settings. A promising alternative approach based on computer vision was demonstrated by Jorquera-Chavez et al. (2021). The advantages of such an approach are its non-invasive nature and the possibility for continuous and automatic monitoring, which may enable more comprehensive solutions.

Knowledge of the daily variation of the animals’ body weight in real-time would allow farmers to improve the animals’ well-being and production. This information can be used to improve nutritional management practices, predict and control the weight at slaughter, and, potentially, serve as a monitor for disease outbreaks (Brandl and Jørgensen, 1996; Kashiha et al., 2014) and thermal stress (Black and Banhazi, 2013).

Weighing animals is a laborious and invasive process. Many attempts to find alternative approaches that would allow for continuous body weight monitoring have had varying success. Automatic scales (Slader and Gregory, 1988; Ramaekers et al., 1995; Schofield et al., 2002) can be successfully used for individual continuous monitoring of older pigs. For younger animals, the most feasible method of daily body weight monitoring in commercial settings explores the correlation between body weight and body dimensions. Some of these methods, such as tapes and calipers, have been widely used by producers. Although these are faster methods than manual weighing, they are not automatic methods and would hinder the development of precision technologies system. Alternatively, several authors (Schofield et al., 1999; Whittemore and Schofield, 2000; Wang et al., 2008; Kashiha et al., 2014; Condotta et al., 2018a; Jun et al., 2018; Pezzuolo et al., 2018; Fernandes et al., 2019) have developed techniques for obtaining animals’ dimensions from digital color and depth images, and this has been shown to be an efficient non-invasive method for body weight prediction. More recently, machine learning techniques for body weight estimation based on images have also shown good results (Condotta et al., 2018b; Suwannakhun and Daungmala, 2018; Cang et al., 2019). Overall, body weight prediction through computer vision seems to be the most promising technique.

### Behavioral measurements

Feed intake, feeding behavior, and water usage are valuable indicators of the health status of pigs (Brown-Brandl et al., 2013; Kashiha et al., 2013). Feed intake and feeding behavior can also serve as indicators of thermal conditions (Black and Banhazi, 2013; Cross et al., 2020). Most automatic individual feeding intake and feeding behavior systems studied for swine involve the use of electronic feeders and/or radio frequency identification antennas (Brown-Brandl et al., 2013; Andretta et al., 2016). However, for younger piglets, a computer vision approach would be more suitable to automatically and continuously assess their feeding behavior, which includes nursing behavior. Computer vision-based machine learning algorithms have been shown to accurately detect pigs’ feeding and nursing behavior (Yang et al., 2018; Yang et al., 2019) and have great potential to be used in a continuous, real-time management system.

A pig’s physiological state can be inferred by certain behaviors, like reduced activity level can be an indication of sickness and huddling a sign of thermal stress and febrile state (Mount, 1960; Ahmed et al., 2014; Matthews et al., 2016; Cook et al., 2018). Behavioral activity is usually acquired through direct observation or the human analysis of video recordings. These methods can be time-consuming and labor-intensive, and their reliability can be affected by the human presence during data collection, which modifies the pigs’ behavior. Continuous recording of pig activity can be achieved using wearable sensors such as accelerometers (Chapa et al., 2020) and radio frequency identification tags (Kapun et al., 2020). However, activity alone is not enough to classify pig behavior and social interactions accurately. A computer vision system for automated assessment of pig activity has been shown to highly correlate with human observations (Ott et al., 2014). Several studies (Ahrendt et al., 2011; Nasirahmadi et al., 2015; Lee et al., 2016; Liu et al., 2020) have demonstrated that computer vision is a promising technique to provide reliable, continuous, and automated behavioral and activity information for young pigs. This technique seems to be the most suitable for the behavioral recognition of young pigs.

### Environmental measurements

Data on the microenvironment in which the piglets exist is a crucial part of an automated management and monitoring (Figure 1) system. Continuous, automatic, and real-time information on the environment can be acquired through sensing technology, such as dataloggers with cloud-gateway connectivity and several modern ventilation control systems. Air temperature, relative humidity, mean radiant temperature, and airspeed are the most commonly monitored environmental variables. Those, along with information on equipment and crate design and nutrition provided to the animal, make up the animal microenvironment that should be modified according to the feedback provided by the animal bioresponses to such environment. When the animal is a biosensor, which has been shown (Sartor et al., 2021), reliable information can be determined about its environment. Real-time animal behavioral analysis might prove to provide a better characterization of the housing environment than the most commonly used combination of electronic sensors and animal comfort indices, which fail to be updated at the same rate as changes in animal genetics, nutrition, and management practices happen.

## Conclusions

This comprehensive review summaries and synthesizes the critical aspects of the environment surrounding piglets through their first 8 wk of life. This includes, farrowing, the creep area, transportation from farrowing, and placement in new housing for growing or finishing. These piglets must overcome being wet at birth, a high surface area to body weight ratio, poor insulation, low vasomotor control, and minimal metabolic activity. Due to these limitations, key considerations include husbandry practices to dry piglets, promote access to heat, and carefully consider the impact of flooring on thermal comfort. This review provides the current knowledge on this piglet-environment interaction and approaches to minimize the environment as a stressor.

The pre-weaning phase requires a warm microclimate, especially during the first 12 h. Caretaker interventions at birth can mitigate the extent and impacts of hypothermia for piglets; albeit, low birth weight remains a dominant risk factor for mortality throughout the pre-wean phase. Piglet mortality is a complex, multidimensional problem and additional large-scale field studies are needed to further evaluate the impacts of active interventions on preweaning mortality and productivity, at birth as well as throughout the entire lactation period.

During the weaning phase metabolic heat production can drop due to limited feed intake. This creates challenges with thermoregulation due to low basal heat production, limited heat increment, and a reduction in fat deposits which act as insulation when lower temperatures are experienced. The combination of relative humidity, airspeed, flooring type, and stocking density impact how pigs experience the thermal environment and should be considered when selecting set-point temperatures and other husbandry practices. Transport from farrowing to either a nursery or wean-finish barn also creates stress during this challenging time. These pigs are particularly susceptible to thermal stress and a reduction in long transport times during extreme weather conditions (hot or cold) should be targeted to reduce transport mortalities.

Numerous technologies have been developed to monitor and characterize the physiological and behavioral responses of young piglets, with varying degrees of success. Contact-based or invasive animal-based measures are challenging for young pigs due to their size, social dynamics, and environment. As increased information regarding the response of young pigs to different thermal and air quality (i.e., carbon dioxide, ammonia, dust, etc.) environments becomes of greater interest, integration of more robust, real-time electronic air quality sensors in farrowing and nursery will be essential. Future control of the environment will integrate real-time animal behavior (physiological analysis) to provide a better characterization of the housing environment than the commonly used sensors and animal comfort indices.
